# All-Cellulose Composites: A Review of Recent Studies on Structure, Properties and Applications

**DOI:** 10.3390/molecules25122836

**Published:** 2020-06-19

**Authors:** Behnaz Baghaei, Mikael Skrifvars

**Affiliations:** 1 Department of Textile Technology, Faculty of Textiles, Engineering and Business, University of Borås, SE-501 90 Borås, Sweden; 2 Department of Resource Recovery and Building Technology, Faculty of Textiles, Engineering and Business, University of Borås, SE-501 90 Borås, Sweden; mikael.skrifvars@hb.se

**Keywords:** all-cellulose composites, fiber/matrix bond, mechanical properties, solvent, antisolvent

## Abstract

Nowadays, there is greater demand for greener materials in societies due to environmental consciousness, depleting fossil fuels and growing ecological concerns. Within the foreseeable future, industries and suppliers will be required to be more aware of challenges faced due to the availability of resources and use more sustainable and renewable raw materials. In this context, cellulose can be expected to become a vital resource for materials owing to its abundance, versatility as a biopolymer, several different forms and potential applications. Thus, all-cellulose composites (ACCs) have gained significant research interest in recent years. ACC is a class of biocomposites in which the matrix is a dissolved and regenerated cellulose, while the reinforcement is undissolved or partly dissolved cellulose. This review paper is intended to provide a brief outline of works that cover recent progress in the manufacturing and processing techniques for ACCs, various cellulose sources, solvents and antisolvents, as well as their properties.

## 1. Introduction

Fiber-reinforced polymer composites have been applied in numerous applications for several years and the market is growing unceasingly. There are challenges related to the polymer composite after its service life, such as due to the nature of the two constituents (the reinforcement and the matrix), reuse and recycling are rather difficult. Consequently, its end-of-life treatment is often carried out by incineration or landfill disposal [1,2]. These days, the demand for environmentally friendly materials is considerable because of increased environmental awareness; as a result, the interest in biocomposites has grown enormously [3,4]. Composite materials made from natural/bio fiber and petroleum-based plastic or renewable resource-based plastic, as well as those produced from synthetic fibers and biopolymers, could also be called biocomposites. Those derived from plant-derived fibers, together with bio-derived plastic, are likely to be more eco-friendly [5,6]. These 100% biocomposites are attractive because they are sustainable and environmentally friendly. At the end of their life, they can easily be disposed or composted without negative impacts on the environment; moreover, they can be recycled by energy recovery. What makes them stand out is that they are designed with the lowest possible environmental footprint [7].

Table 1 provides a list of some of the biodegradable matrices (natural and synthetic) that can be applied for making biocomposites [8,9].

In recent years, a great number of studies have been devoted to biopolymer composite materials reinforced by natural or man-made cellulosic fibers [3,4,10,11,12]. The most broadly used biopolymers for the development of biocomposites are poly(lactic acid) (PLA), cellulose esters, polyhydroxyalkanoates (PHAs) and starch-based plastics [13,14,15]. The commercial applications of biocomposites are increasing in different industrial sectors, mostly in transport and automotive applications, based on some merits including low production cost due to their universal availability, greater modulus/weight ratio compared to E-glass fibers, reduced risk of environmental pollution, etc. [16]. On the other hand, biocomposites have some setbacks which limit their structural applications in certain engineering components. One of the main challenges with biocomposites is the poor interfacial adhesion between hydrophobic matrices and hydrophilic reinforcing fibers, often resulting in lower mechanical properties of the composite, high water uptake and fiber swelling [16,17]. Following the eco-design concepts, self-reinforced polymer composites or single polymer composites could be another alternative route to environmentally friendly polymer composites that overcome this incompatibility. In 1975, Capiati et al. proposed high density polyethylene composites, which possessed a gradually changing morphology between the matrix material and reinforcing fibers, to substitute conventional glass fiber reinforced composites in several applications [18]. In 2004, Cabrera et al. produced all-polypropylene composites, namely polypropylenes (PPs) reinforced with oriented polypropylene fibers, to substitute glass fiber for use in applications in the automotive industry due to their recyclability and eco-friendly nature [19]. These all-polypropylene composites were based on the combination of PP fibers having a higher melting point than the matrix PP.

## 2. All-Cellulose Composites

A combination of the current trends towards natural/bio fiber reinforcement and self-reinforced polymer composites has recently led to studies on the development of all-cellulose composites (ACCs). The ACC concept was first proposed by Nishino et al. [20]. ACCs are manufactured solely from cellulose, which functions both as the incorporated fiber reinforcement and the matrix.

### 2.1. Cellulose as a Biopolymer

Among all of the biopolymers, cellulose, a polymer of β(1→4) linked glucose monomers, is the most abundant natural resource on the planet with an annual yield of nearly 1.5 trillion tonnes, and is an almost inexhaustible source of raw material in the manufacturing of environmentally friendly and sustainable bioproducts [21]. Cellulose can commonly be obtained from plant and algal cell walls and from bacteria that also produce this biopolymer [22]. Regardless of the cellulose resource, cellulose is generally a highly crystalline and high molecular weight biopolymer that has a strong tendency to form high crystalline fibers. However, depending on the resource and applied treatment method during its extraction and regeneration, the degree of polymerisation (DP), fibrillary organisation and crystallinity differ [21]. It appears that at least four different polymorphs of cellulose exist, which are named cellulose I, II, III and IV [22]. Native cellulose, or cellulose I, is the highest crystallinity type, containing two co-existing crystal phases, cellulose I_α_ and cellulose I_β_. The crystal structure of cellulose I is converted to that of cellulose II by sodium hydroxide treatment (mercerization) or by regeneration of cellulose I in the viscose process [22]. Cellulose III, which is obtained by diamine treatment of cellulose I or cellulose II, is designated as cellulose III_1_ or cellulose III_2_, correspondingly [23]. Cellulose IV is formed by heat treating cellulose III in glycerol [24]. The elastic modulus (E_l_) of the crystalline regions of the various cellulose polymorphs in the direction of the chain axis are different, indicating that the polymer skeletons of these polymorphs are entirely different from each other. This is because during the crystallisation transition, the skeletal conformations and intramolecular hydrogen bonds are changed. The E_l_ values of cellulose I, II, III and IV are reported to be 138, 88, 87 and 75 GPa, respectively [25], while the E_l_ value of E-glass is 78.5 GP. This shows that the mechanical properties of cellulose compete well with glass fibers [26].

### 2.2. Cellulose as a Reinforcement

The desirable properties of cellulose, including biocompatibility, high thermal stability, high tensile strength and high modulus, make cellulose a very versatile and important material [27,28]. Cellulose is used in a wide range of applications including in the food and pharmaceutical industries, smart materials, packaging, coatings, upholstery, etc. Furthermore, owing to the excellent mechanical properties of cellulose, it is extensively used as a bio-based reinforcement in composites [29,30,31,32,33]. Cellulose as reinforcement has been discussed and presented in many review papers [34,35,36]. Wood fibers, as the main source of cellulose fibers, are extensively used and investigated in composites. Several polymers can be applied as a matrix material, such as high density polyethylene, low density polyethylene, polypropylene, polyvinyl chloride and ethylene vinyl acetate copolymer [37,38,39,40,41,42,43,44,45,46,47]. Besides wood fibers, polymer composites reinforced with other natural fibers such as kenaf, hemp, flax and jute have been widely studied [11,48,49,50,51,52,53,54,55,56,57,58].

### 2.3. Need for ACCs and Benefits

ACCs are becoming increasingly popular because of their significant properties such as biocompatibility, biodegradability, nontoxicity and renewability of the raw materials. As the composite is composed of only cellulose, recycling is facilitated. ACCs are fully biodegradable after their service life. Being environmentally friendly and sustainable are the major attractions for ACCs. ACCs are suited for both disposing or composting at the end of their life without damaging the environment, or can be recycled by energy recovery [59,60].

In comparison to the conventional composites used today, ACCs can provide distinct advantages. The primary advantage is the almost perfect chemical bonding at the reinforcement–matrix interface, which is the key driver for the development of ACCs. The matrix and the reinforcement are chemically identical and fully compatible with each other, allowing for efficient stress transfer and adhesion at their interface [61,62,63].

Moreover, the concept of ACCs also allows for the manufacturing of composites with higher reinforcement content (non-dissolved cellulose fibers) than for traditional fiber-reinforced composites [64]. Soykeabkaew et al. [65] produced ACCs using a surface selective dissolution method. In this study, Bocell fibers were partially dissolved, resulting in volume fractions of up to 90% of the original fiber and 10% of newly regenerated cellulose matrix. The ACCs exhibited an average tensile strength of 910 MPa and a Young’s modulus of 23 GPa with 8% elongation at break, which are significantly higher in comparison to the traditional unidirectional natural fiber reinforced composites reported in previous studies [66,67,68,69,70,71,72,73]. 

Furthermore, the mechanical, optical and barrier properties of ACCs are significantly better than those of common cellulose-based materials [60]. Yousefi et al. [74], manufactured ACCs from cellulose microfibers as a raw material via solvent-based nanowelding. In producing the ACCs, the nanowelding process connected the structures together and made an extended network of reinforcement–matrix shared by adjacent nanofibrils. This connectivity improved the structural integrity of the ACCs which resulted in good stress transfer through the continuous network of reinforcement–matrix. Air permeability measurement showed that the cellulose microfiber sheet had a structure highly permeable to air (42 ± 7 μm Pa^−1^·s^−1^), while ACCs formed a complete barrier protection to air (0 μm Pa^−1^·s^−1^) like conventional packaging polymers. The superior barrier quality of ACCs compared to the cellulose microfiber is attributed to their fully consolidated structure. In addition, the obtained ACCs showed high transparency (76% at a wavelength of 800 nm) compared to a microfiber sheet (0.3%) which is about a 250-fold increment. The high transparency of ACCs can be attributed to the thinner reinforcements during the partial dissolving/nanowelding of the microfibers to make ACCs [75] which results in a more effective interface, low void volumes and less surface roughness [75,76] compared to those of cellulose microfiber sheets.

### 2.4. Fabrication Methods

**Figure 1:**
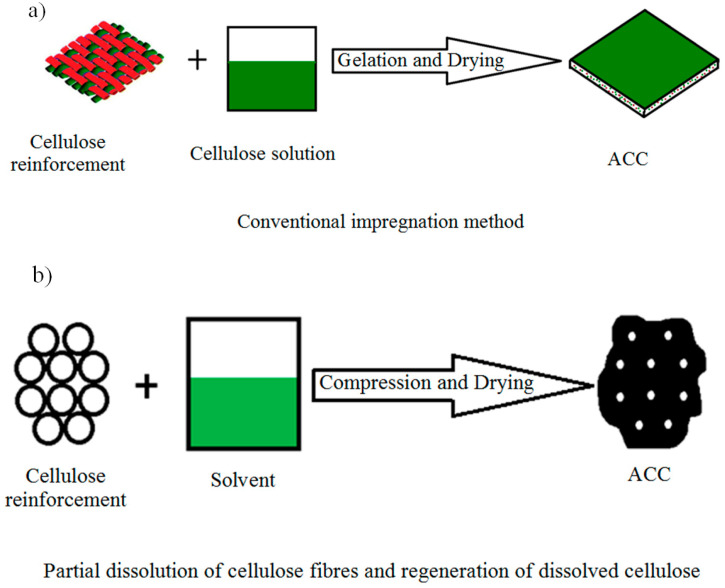
Schematic of processing approaches of all-cellulose composites (ACCs): (**a**) conventional impregnation method (CIM) and (**b**) partial dissolution (PD) method.

Two different processing approaches have been demonstrated for the manufacturing of ACCs: (i) the complete dissolution of a cellulose material with an appropriate solvent followed by conventional impregnation methods (CIM) of fibrous cellulose material, which will act as the reinforcement (Figure 1a) [62], and (ii) partial dissolution (PD) of a cellulose fabric to form a matrix phase that bonds undissolved cellulose fiber cores together (Figure 1b) [77]. Gindl and Keckes [77] were the pioneers of the latter method, and it has been variously referred to as partial dissolution [78], surface-selective dissolution [79] or natural fiber welding [80,81].

Despite the fact that both processing approaches are possible, the PD method seems to be more realistic concerning a potential industrial upscaling [81]. This is due to the fact that the total differential shrinkage, which results in dimensional instability and internal residual stresses, is quite a big challenge while producing ACCs, and the triggered shrinkage is generally lower for the ACCs made via the PD method than the CIM [78,82]. Most researchers have manufactured and characterized films of ACCs with thicknesses that are <1 mm. Nevertheless, the development of ACCs into diverse applications will undoubtedly require greater thicknesses of the material. Furthermore, Duchemin reported problems associated with differential shrinkage that results in lateral shear stresses in the dimensionally thin laminates. The shrinkage is due to the water diffusion processes during composite manufacturing and the strong hydrophilicity and water uptake of the cellulosic material [83]. Huber et al. [78] developed a new approach termed solvent infusion processing with the aim of manufacturing thick (>1 mm) ACC laminates.

### 2.5. Different Solvents

ACCs have attracted great interest lately, and several researchers have been working on this exciting topic. A number of different cellulose types, cellulose solvents and anti-solvents have been applied to process ACCs. The most commonly used solvents are *N*-methylmorpholine-*N*-oxide (NMMO), lithium chloride/*N*,*N*-dimethylacetamide (LiCl/DMAc), ionic liquids (ILs), and sodium hydroxide (NaOH) aqueous solution [63], of which LiCL/DMAc and ILs have so far been the most favored and efficient solvents with high capacity for dissolving high molecular weight cellulose [84], while the choice of cellulose source for matrix and reinforcement has been more open.

### 2.6. Cellulose Sources

Though this review covers different cellulose sources as reinforcement and matrix for ACCs, it will definitely be incomplete due to extensive research in this field; however, the aim is to provide a practical overview of a wide range of cellulose resources used for ACCs. Table 2 presents different properties of ACCs fabricated by different processes, cellulose resources, solvents and anti-solvents.

The choice of cellulose source mostly defines the type of the obtained ACCs. Isotropic ACCs have been produced from micro- or nano-fibrillated cellulose [77,82,85,86,87], bacterial cellulose [88], wood pulp [89] and filter paper [85], while non-isotropic ACCs including uni- and multi-directional ones have been manufactured from bio/natural fibers such as ramie [79,90,91], alfa [92], cotton ([93]), and flax [84], etc. and regenerated cellulose fibers including Cordenka [78,94], Lyocell [84] and Bocell [65].

### 2.7. The Role of Anti-Solvent in Cellulose Regeneration

**Figure 2:**
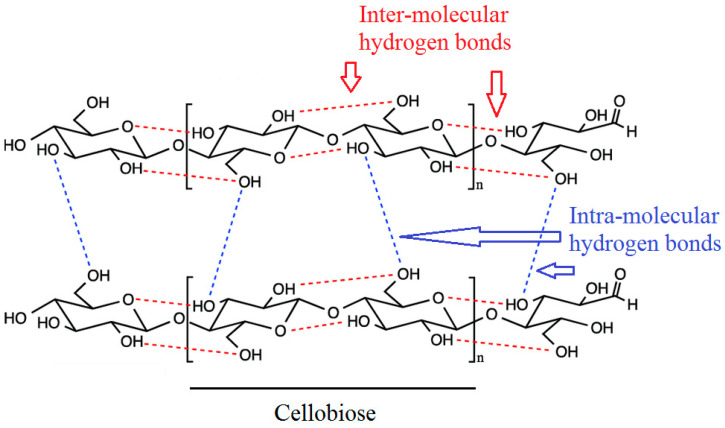
Cellulose structure.

It is well known that cellulose regeneration is an important step in manufacturing ACCs. The cellulose molecular chains are composed of inter- and intra-molecular hydrogen bond networks (Figure 2) where the higher hydrogen basicity of the solvents can weaken the hydrogen bonds of the cellulose, leading to dissolution of cellulose [95]. For regenerating cellulose dissolved in ILs, it is typically necessary to add anti-solvents (a coagulation medium) such as water, ethanol, acetone, methanol or acetonitrile into the cellulose solution in order to precipitate the cellulose [96,97,98]. By exchanging the solvent with an anti-solvent, inter- and intra-molecular hydrogen bonds between the hydroxyls are reestablished, which results in the precipitation and regeneration of the dissipated cellulose chains [99,100,101]. The structure, mechanical, thermal and surface chemical properties of the regenerated cellulose depend largely on the type of cellulose solvent system and anti-solvent due to the irregular motion of the macromolecular chains and the establishment of intra- and inter-molecular hydrogen bonds in cellulose during regeneration [81,102,103]. The water molecules form hydrodynamic shells around the ionic liquid molecules, inhibiting the direct interactions between cellulose and ionic liquid molecules. Further, the ionic liquids can be recovered by vacuum evaporation [98]. Depending on the requirements for the final product, different regeneration processes are designed, leading to different forms such as films, beads, gels, etc. [96].

Recently, the regeneration process of cellulose from LiCl/DMAc solution [104], the aqueous alkali/urea system [105], 1-butyl-3-methylimidazolium acetate [106], 1-butyl-3-methylimidazolium chloride [107] and 1-allyl-3-methylimidazolium chloride [108] have been broadly studied. Tan et al. [109] provided a detailed investigation about the effect of different anti-solvents (water, ethanol, or combinations of water and ethanol) on the characteristics of cellulose that is dissolved and then generated from 1-ethyl-3-methylimidazolium acetate. The crucial role of anti-solvent in controlling the structure and properties of the regenerated cellulose was reported. They observed that when ethanol is added to the cellulose–IL solution, it can interact with the anion of IL by hydrogen bonding, which leaves the cellulose chains twined and aggregated. Then, these cellulose chains can rearrange themselves into ordered regions with a fairly loosened aggregated structure and fewer tendencies to contribute to forming cellulose crystallites, which result in a more amorphous regenerated cellulose material. When water is used as an anti-solvent, it can simply penetrate into the cellulose–solvent solution system and react with both the anion of the IL and the cellulose hydroxyl groups via hydrogen bonding, which leads to easier aggregation and realignment of cellulose molecular chains and the formation of cellulose crystallites. Moreover, since the polarity of a water molecule is greater than an ethanol molecule during the regeneration process, forming hydrogen bonds with cellulose is easier with water. Consequently, regeneration in water allows for the formation of more oriented and crystalline structured cellulose.

Elhi et al. [110] manufactured electrically conductive composite materials using an IL as the solvent, cellulose as the binder and carbon aerogel as the conducting material. Regeneration of cellulose and composites from ILs was carried out using different anti-solvents including water, ethanol and acetone. They observed that among these anti-solvents, water showed the best regeneration properties, i.e., it could regenerate cellulose and dissolve ILs from the composite. They reported that water regenerates more cellulose from IL as compared to acetone and ethanol as it forms more hydrogen bonds with IL ions. Since ethanol and acetone are larger molecules than water, they face more steric hindrance when moving between cellulose polymer chains. Moreover, since IL anions break the hydrogen bonds in cellulose, they are more intensely attracted to water molecules with their stronger hydrogen bonds than ethanol and acetone molecules.

### 2.8. ACCs Overview

A general assessment and review of the potential mechanical properties of ACCs are very challenging since they are influenced by several factors such as cellulose resource, type of reinforcement, manufacturing process conditions, etc. [81]. The immersion time in the solvent is one of the foremost parameters in the production of ACCs, which has a noticeable effect on the formed microstructure and crystallinity of ACCs, as well as the degree of polymerization of the processed cellulose [79,83]. Soykeabkaew et al. manufactured ACCs via a partial dissolution method of aligned ramie fibers and investigated the effect of the dissolution time on the structure, as well as the thermal and mechanical properties of ACCs. They reported that by increasing immersion time of the fibers in the solvent, a higher fraction of the fiber skin is transformed into the matrix phase. This leads to a decrease in the longitudinal tensile strength due to the reduction of the fiber cross-sectional area. At the same time, there is an improvement in transverse tensile strength, due to a higher matrix volume fraction and stronger interfacial interaction [79]. Another important parameter is the cellulose regeneration rate (precipitation rate). Duchemin et al. produced ACCs via partial dissolution of microcrystalline cellulose in LiCl/DMAc solution under both a fast and slow precipitation route. They reported that the mechanical properties and final morphology of ACCs are governed significantly by the dissolution time, cellulose concentration and regeneration time. ACCs prepared by slow precipitation showed higher crystallinity and improved tensile properties [82].

**Figure 3:**
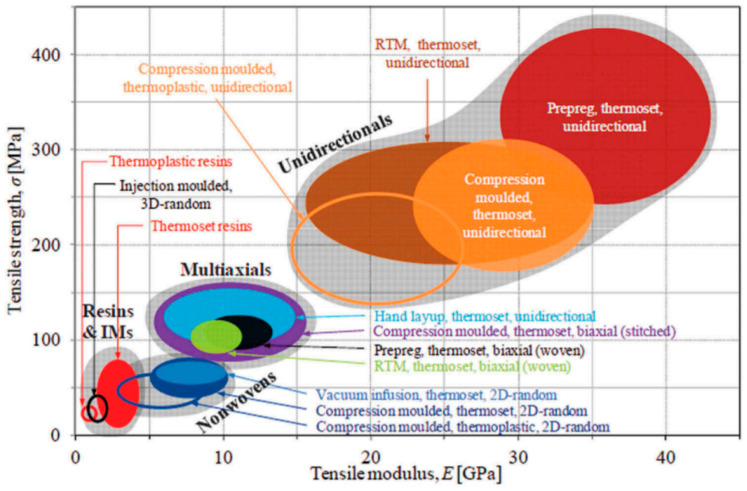
Typical tensile properties for plant fiber reinforced plastics manufactured with thermoplastic/thermoset resins, short-random/long-aligned fiber reinforcements, and various manufacturing processes (reproduced from [145]). RTM = resin transfer moulding, IM = injection moulding.

**Figure 4:**
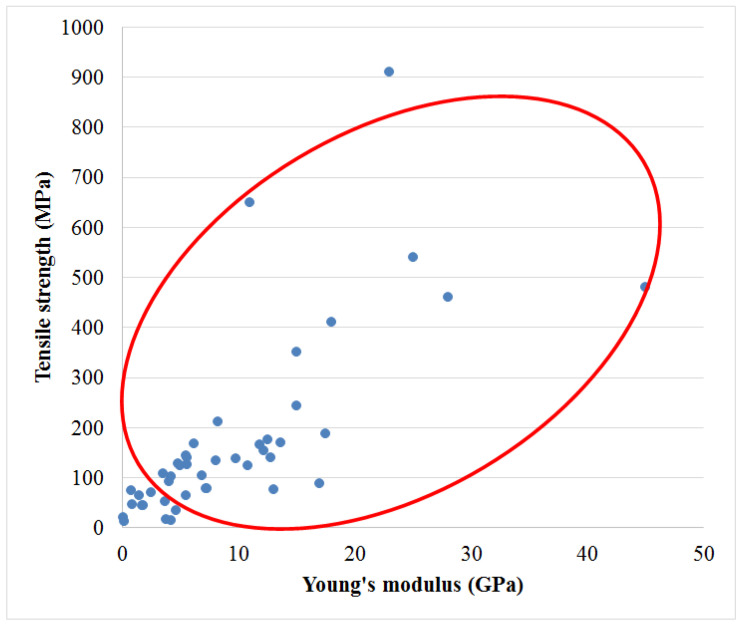
Mechanical properties of ACCs (tensile versus mechanical strength). The plot has been produced using data presented in Table S1.

Table 2 lists the mechanical properties of ACCs manufactured using different materials, solvents, anti-solvents and processes. Tensile, flexural and impact properties have been reported and are described in the table. Figure 3 presents an Ashby plot for different plant fiber reinforced plastics, illustrating the range in tensile stiffness and strength. An overview of the mechanical properties of ACCs is presented in Figure 4. A comparison of ACCs with other plant fiber reinforced plastics shows that ACCs are greatly competitive in terms of their properties. Several studies have been conducted to examine the possibility of realizing ACCs with enhanced final mechanical properties. Most of this research shows that it is possible to achieve various degrees of improvement in tensile properties with the manufacture of the respective ACCs. A number of ACCs have been prepared by different techniques, and a variety of solvents have the ability to dissolve cellulose. The most widely used and efficient solvents to investigate ACCs are ILs (particularly 1-butyl-3-methylimidazolium chloride (BMIMCl)), LiCl/DMAc and sodium hydroxide with additives. Most of these solvents have limited potential for application on an industrial scale on account of toxicity, non-recyclability and slow dissolution time [63]. LiCl/DMAc has commonly been applied in research that followed the partial dissolution (PD) ACC manufacturing method. By applying LiCl/DMAc, the amount of selective dissolution of cellulose can be controlled by the pre-treatment step which forms part of the solvent system [20]. Unfortunately, the use of this solvent is limited to academic research due to environmental concerns [85]. Ionic liquids have attracted attention as a green solvent for ACC production due to their positive properties including thermal stability, easy recyclability, negligible vapor pressure and high capacity to dissolve a wide range of cellulose without activation or pre-treatment [35].

For ACC production, both natural cellulose and different regenerated cellulose can be applied as the raw material. Natural cellulose mostly contains cellulose I. Cotton fiber is one of the natural celluloses with high cellulose content. ACCs based on cotton fiber reported by Arevalo et al. [111] achieved a tensile strength of 144 MPa. Compared with cotton fibers, most natural plant fibers have a lower content of cellulose and higher contents of hemicellulose and lignin. When choosing a cellulose raw material, high cellulose purity is essential to avoid contamination of the IL solvent with decomposed hemicellulose and lignin fractions since lignin and hemicellulose are also soluble in ILs [112]. The existence of lignin and hemicellulose can act as impurities which decrease the mechanical properties of ACCs. In order to solve this problem, different pre-treatments can be performed to eliminate the hemicellulose and lignin. Yousefi et al. managed to fabricate an all-cellulose nanocomposite [74]. Highly purified cellulose fibers were directly welded at the nanoscale using DMAc/LiCl solvent. To purify cellulose, canola straw was first treated with sodium hydroxide/anthraquinone, followed by sequentially treating with chloride dioxide, NaOH/hydrogen peroxide and ClO_2_ which is an ordinary bleaching process. In order to increase cellulose purification, an excessive bleaching process was conducted via further treating the cellulose fibers with potassium hydroxide and sodium chloride. In this step, all impurities including lignin and the hemicelluloses were removed completely. Finally, the manufactured ACC showed an enhanced tensile strength and Young’s modulus of 188 MPa and 17.5 GPa respectively. Qin et al. investigated the effect of mercerization or alkali treatment on the properties of the prepared ACCs from dissolved ligno-cellulosic ramie fibers in LiCl/DMAc [91]. The treatment improved the tensile strength of the prepared ACCs by 15–95%. The positive effect of mercerization on cellulosic fibers (specifically an improvement of tensile properties) could be due to that during mercerization, a swelling of the reinforcing fibers leads to filling of the cracks and voids between them. Accordingly, the fibers will be merged together, resulting in a significantly improved interface and tensile properties of the mercerized composite [113,114].

Regenerated cellulose fibers mostly consist of cellulose II. One of the most important parameters that makes regenerated fibers favorable in ACC production on a commercial scale is their uniformity in properties and shapes between different batches [60,78,94,115,116,117,118]. Adak et al. [117] manufactured Lyocell-based ACCs via a compression molding technique using IL (1-butyl-3-methylimidazolium chloride) as a solvent. ACCs show a maximum tensile strength of 102 MPa, which is significantly higher than that of flax-based ACCs (46 MPa [119] and 34 MPa [84]). Currently, the impact and flexural resistance of ACCs are principally unknown since most of the studies performed on ACCs are focused on evaluating the tensile properties. According to the findings reported from previous research, flexural strength and modulus of ACC laminates range between 48.9–178 MPa and 0.96–11 GPa, respectively. Huber et al. [94] produced Cordenka-based ACCs by partial dissolution of the textiles, followed by vacuum infusion. Flexural properties and fracture behavior of ACCs during impact loading were investigated. The produced ACCs exhibited relatively high impact strength. The response of the ACCs to Charpy impact testing displayed two different failure modes: fiber failure and splitting of the matrix connecting individual fibers and fiber bundles. In the ACCs, all fibers are surrounded by a continuous cellulosic matrix; consequently, before fracturing the fibers, the crack propagates through the matrix phase which results in good impact properties. Furthermore, the high strain to failure of regenerated cellulose fibers also contributes to the higher impact strength compared to biocomposites based on natural bast fibers. It is also reported that the flexural strength of the ACCs is superior to many other biocomposites, which could be due to the strong interfacial adhesion present in these materials. Moreover, Liu et al. [120] and Shakeri et al. [121] studied the dynamic mechanical thermal properties of different ACCs.

## 3. Future Scope of ACCs and Application

ACCs are used in a wide range of applications such as structural materials, biomedical engineering (substitution of bone and cartilage materials), photoelectric devices, electro-active paper, sensors, electrical displays, filtration materials, biodegradable food packaging materials and mulching films for agriculture [60,76,79,135,143,146,147,148,149,150,151,152,153,154].

The rapid biodegradation of ACCs is a key advantage over other biocomposites, which however conversely restrict the use of ACCs in some applications [59]. Furthermore, the strong hydrophilicity and water uptake are the main drawbacks of ACCs, which limits the outdoor applications of ACCs. Several methods have been investigated to decrease the hydrophilic character of the cellulose surface [155,156,157,158]. Alkyl-based or fluorine-based silane coupling agents have been found to be effective due to their high water resistance and ability to couple hydrophilic and hydrophobic functional groups. Surface treatments or bulk modifications are probably needed to protect the mechanical properties of ACCs while exposed to moisture. Yousefi et al. produced water-repellent ACCs using the silane coupling agent dodecyltriethoxysilane (3 wt%). The ACCs consist of cellulose nanofibers incorporated into a cellulosic matrix. They found that immersion treatment of ACCs with a silane coupling agent results is an increase in water contact angle from 59° to 93° and decreases the water uptake from 4% to 1%. During environmental exposure, the potential damage connected to moisture, including deformation, fungal decay and weakening of mechanical properties, can be diminished for treated ACCs [159].

## 4. Conclusions

The use of petroleum-based polymers for conventional composite production has initiated a global social concern due to the pollution derived from their synthesis and the related littering problems. Therefore, bio-based composite materials are attracting the attention of researchers. The principal motivation for developing biocomposites is to make a new generation of composites which are environmentally compatible in terms of manufacturing, application and recycling. Cellulose is the most abundant biopolymer on Earth and one of the most promising bio-renewable resources for reducing and replacing the massive amount of petroleum-based plastic materials. Among the large group of biocomposite materials, all-cellulose composites (ACCs) are a category of specific interest. Both the reinforcement and the matrix in ACCs are cellulose, which improves the compatibility between the phases and consequently the mechanical properties of the composite. Different sources of cellulose and their composite properties have been investigated. Furthermore, the effects of different processing conditions and different solvents as well their ACCs have been discussed in detail. The recent development of ACCs can open up new opportunities for both academics as well as industries to generate new applications for ACCs. A key advantage of ACCs over other biocomposites is the rapid biodegradation using a soil burial bed, but the great potential for degradation correspondingly limits the use of ACCs in some applications.

## Figures and Tables

**Table 1:** Biodegradable polymer matrices (adapted and developed from [9]).

Biodegradable Polymers	
Natural	Synthetic
Polysaccharides: Starch, Cellulose, Chitin Proteins: Collagen/Gelatin, Casein, Albumin, Fibrinogen, Silks Polyesters: Polyhydroxyalkanoates Other polymers: Lignin, Lipids, Shellac, Natural Rubber	Poly(amides) Poly(anhydrides) Poly(amide-enamines) Poly(vinyl alcohol) Poly(vinyl acetate) Polyesters: Poly(glycolic acid), Poly(lactic acid), Poly(caprolactone), Poly(orthoesters) Poly(ethylene oxides) Poly(phosphazenes)

**Table 2:** Literature review on precedents of ACC production.

Reinforcement	Matrix	Solvent	Anti-Solvent	Process	Mechanical Properties												Ref.
Ramie	Kraft pulp from coniferous trees	LiCl/DMAc	Methanol	CIM	Young’s modulus (GPa)						Tensile strength (MPa)						[20]
					45						480						
Microcrystalline cellulose	-	LiCl/DMAc	Distilled water	PD	Young’s modulus (GPa)						Tensile strength (MPa)						[77]
					15						243						
Beech pulp fibers	-	LiCl/DMAc	Distilled water	PD	Young’s modulus (GPa)						Tensile strength (MPa)						[89]
					12.2						154						
Filter paper	-	LiCl/DMAc	Methanol	PD	Young’s modulus (GPa)						Tensile strength (MPa)						[90]
					8.2						211						
Ramie	Ramie	LiCl/DMAc	Methanol	CIM	Young’s modulus (GPa)						Tensile strength (MPa)						[91]
					25						540						
Ramie	-	LiCl/DMAc	Methanol	PD	Young’s modulus (GPa)						Tensile strength (MPa)						[79]
					28						460						
Micro-fibrillated cellulose and filter paper	-	Ionic liquid: 1-butyl-3-methylimidazolium chloride	Water	PD	10.8						124						[85]
Native cellulose nanowhiskers	-	NaOH/urea	Distilled water	PD	Young’s modulus (GPa)						Tensile strength (MPa)						[122]
					5						124						
Microcrystalline cellulose	-	LiCl/DMAc	Distilled water	PD	Young’s modulus (GPa)						Tensile strength (MPa)						[82]
					6.9						105						
Lyocell Bocell	-	N-methyl morpholine N-oxide (NMMO)	Methanol	PD	Young’s modulus (GPa)						Tensile strength (MPa)						[65]
					Lyocell: 15 Bocell: 23						350 910						
Bacterial cellulose	-	LiCl/DMAc	Methanol	PD	Young’s modulus (GPa)						Tensile strength (MPa)						[88]
					18						410						
Rice husk	Filter paper	Ionic liquid: 1-butyl-3-methylimidazolium chloride	Water	CIM	Young’s modulus (GPa)						Tensile strength (MPa)						[123]
					17						89						
Microcrystalline cellulose	-	DMAc/LiCl	Distilled water	PD	Young’s modulus (GPa)						Tensile strength (MPa)						[86]
					1.5						65						
Filter paper	-	PEG/NaOH aqueous solution	Water	PD	Young’s modulus (GPa)						Tensile strength (MPa)						[124]
					0.75						74						
Eucalyptus pulp	Softwood dissolving	-	Water	CIM	Young’s modulus (GPa)						Tensile strength (MPa)						[125]
					13						76						
Cellulose nanowhiskers	Wood pulp	-	Water	CIM	Storage modulus at 20 °C (GPa) 4.9												[120]
Cellulose nanowhiskers	Microcrystalline cellulose (MCC)	LiCl/DMAc	Water	CIM	Young’s modulus (GPa)						Tensile strength (MPa)						[126]
					4.8						128.4						
Nanocrystalline cellulose (NCC)	Pretreated microcrystalline cellulose (PMCC)	Ionic liquid: 1-(2-hydroxylethyl)-3-methyl imidazolium chloride (HeMIMCI)	Water	CIM	Young’s modulus (GPa)						Tensile strength (MPa)						[127]
					3.7						52						
Cellulose nanocrystal	Dissolved eucalyptus pulp	NMMO	Water	Co-electrospinning	Young’s modulus (GPa)						Tensile strength (MPa)						[128]
					5.6						140						
Cellulose nanowhiskers	Cotton linter pulp	NaOH/urea	Water	Rapid thermal-induced phase separation	-												[129]
Nanofiber of canola	-	LiCl/DMAc	Methanol	PD	Tensile strength (MPa) 164												[130]
Canola straw	-	Ionic liquid: 1-butyl-3-methylimidazolium chloride (BMIMCl)	Methanol	PD	Young’s Modulus (GPa)				Tensile strength (MPa)				Strain at break (%)				[74]
					17.5				188				11.8				
Cellulose nanocrystals	cellulose acetate	Acetone and DMAc	KOH solution in ethanol	Electrospinning	-												[131]
Microfibrillated cellulose (MFC)	-	Ionic liquid: 1-butyl-3-methylimidazolium chloride (BMIMCl)	Water	PD	Storage modulus at 40 °C (GPa)												[121]
					1.1												
Linen flax fiber Rayon	-	Ionic liquid: 1-butyl-3-methylimidazolium acetate (BMIMAc)	Distilled water	PD	Young’s Modulus (GPa)						Tensile strength (MPa)						[119]
					Linen: 0.86 Rayon: 2.45						46 70.16						
Cordenka	-	Ionic liquid: 1-Butyl-3-methylimidazolium acetate (BMIMAc)	Distilled water	PD	Young’s Modulus (GPa)						Tensile strength (MPa)						[78]
					4						92						
Lyocell Flax	-	Ionic liquid: 1-butyl-3-methyl-imidazolium-chloride	Distilled water	PD	Young’s Modulus (GPa)						Tensile strength (MPa)						[84]
					7.2 4.6						78 34						
Cellulose nanowhiskers	Microcrystalline cellulose	LiCl/DMAc	Water	CIM	Young’s Modulus (GPa)						Tensile strength (MPa)						[132]
					12.5						175.6						
Cordenka	-	Ionic liquid: 1-butyl-3-methylimidazolium acetate (BMIMAc)	Distilled water	PD	Impact strength (kN/mm^2^)				Flexural modulus (GPa)				Flexural strength (MPa)				[94]
					1.96				3.8				140				
Cellulose nanowhiskers	Microcrystalline cellulose	LiCl/DMAc	Distilled water	CIM	Young’s modulus (GPa)						Stress at failure (MPa)						[87]
					13.6						170						
					6.12						53						
Cotton fabric	-	Ionic liquid: 1-butyl-3-methylimidazolium chloride (BMIMCl)	Acetonitrile	PD	Young’s Modulus (GPa)						Tensile strength (MPa)						[93]
					0.05						20						
Tunicate cellulose nanowhiskers	Microcrystalline cellulose	LiCl/DMAc	Water	CIM	Young’s Modulus (GPa)						Tensile strength (MPa)						[133]
		NaOH/urea			LiCl/DMAc system: 11.8						165.4						
					NaOH/urea system: 9.8						137.1						
Cotton linters cellulose	Softwood bleached kraft pulp	NaOH/urea/H_2_O	H_2_SO_4_	CIM	Young’s Modulus (GPa)						Tensile strength (MPa)						[134]
					6.2						167						
Sugarcane bagasse nanofibers	-	LiCl/DMAc	Ethanol	PD	Young’s Modulus (GPa)				Tensile strength (MPa)				Toughness (m N m^−3^)				[135]
					12.8				140				8.07				
Coconut Shell Powder and Microcrystalline Cellulose	-	LiCl/DMAc	Methanol	PD	Young’s Modulus (GPa)						Tensile strength (MPa)						[136]
					0.14						12						
Straw cellulose fiber	Microcrystalline cellulose	LiCl/DMAc	Distilled water	CIM	Tensile strength (MPa)				Flexural modulus (GPa)				Flexural strength (MPa)				[137]
					650				4				140				
Microcrystalline cellulose (MCC)	-	Ionic liquid: 1-ally-3-methylimidazolium chloride (AMIMCl)	Water	PD	Young’s modulus (GPa)						Tensile strength (MPa)						[138]
					8.1						135						
Cellulose nanocrystals	-	LiCl/DMAc	Distilled water and methanol	PD	--												[139]
Halloysite nanotubes		LiCl/DMAc	Distilled water and methanol		Young´s modulus (GPa)				Tensile strength (MPa)				Strain at break (%)				
					5.6				126.2				11.4				
Rayon fiber textile	-	Ionic liquid: 1-butyl-3-methylimidazolium acetate (BMIMAc)	Distilled water	PD	Young´s modulus (GPa) 7.3						Tensile strength (MPa) 77.7						[140]
Pulp from paper making	-	Aqueous zinc chloride (ZnCl2) solvent	Tap water	PD	Young’s Modulus (GPa)						Tensile strength (MPa)						[141]
					5.5						64.9						
Cellulose extracted from empty bunch of palm oil	-	LiCl/DMAc	Water	PD	Elongation at break (%)				Young’s Modulus (GPa)				Tensile strength (MPa)				[142]
					3.07				3.56				109				
Cellulose fibrils extracted from native African Napier grass	Cotton	LiOH/urea	Ethyl alcohol	CIM	Elongation at break (%)						Tensile stress (MPa)						[143]
					12.7–8.6						49.7–76.8						
Lyocell	-	Ionic liquid: 1-butyl-3-methyl imidazolium chloride	Distilled water	PD	Young’s Modulus (GPa) 1.7						Tensile stress (MPa) 45						[144]
Cotton	-	LiCl/DMAc	Water	PD	Young’s Modulus (GPa)						Tensile stress (MPa)						[111]
					5.5						144						
Alfa fibers	Alfa pulp Wood pulp	NaOH/water	Water	CIM	Young’s Modulus (GPa)						Tensile stress (MPa)						[92]
					Alfa ACC = 3.8 Wood ACC = 4.2						16 13.9						
Lyocell	-	Ionic liquid: 1-butyl-3-methylimidazolium chloride (BMIMCl)	Water	PD	Young’s Modulus (GPa)			Tensile stress (MPa)			Flexural modulus (GPa)			Flexural strength (MPa)			[116]
					1.8			44.2			0.96			48.9			
Lyocell	-	Ionic liquid: 1-butyl-3-methylimidazolium chloride	Water	PD	Young’s Modulus (GPa)			Tensile stress (MPa)			Flexural modulus (GPa)			Flexural strength (MPa)			[117]
					4.2	102.6					11				178.3		

## Supplementary Materials

The following are available online, Table S1: Literature survey of reported tensile properties of various ACCs.
