# Supplementary material for: All-Cellulose Composites: A Review of Recent Studies on Structure, Properties and Applications
Source: Molecules. 2020 Jun 19;25(12):2836. doi: 10.3390/molecules25122836 (PMC7356676; doi:10.3390/molecules25122836)
Supplement: Supplementary file 1 [file molecules-25-02836-s001.pdf]

Table 1. Literature survey of reported tensile properties of various ACCs.

Tensile Strength (MPa)	Young's Modulus (GPa)	Sample	Reference
480	45	1	All-Cellulose Composite
243	15	2	All-cellulose nanocomposite
154	12.2	3	Structure and properties of a pulp fibre-reinforced composite with regenerated cellulose matrix
211	8.2	4	All-cellulose composite prepared by selective dissolving of fiber surface
540	25	5	The effect of fibre volume fraction and mercerization on the properties of all-cellulose composites
460	28	6	All-cellulose composites by surface selective dissolution of aligned ligno-cellulosic fibres
124	10.8	7	All-cellulose composites by partial dissolution in the ionic liquid 1-butyl-3-methylimidazolium chloride
124	5	8	Properties of Films Composed of Cellulose Nanowhiskers and a Cellulose Matrix Regenerated from Alkali/Urea Solution
105	6.9	9	Duchemin et al. (2009) (Structure-property relationship of all cellulose composite
350	15	10	All-cellulose composites of regenerated cellulose fibres by surface selective dissolution
910	23	11	All-cellulose composites of regenerated cellulose fibres by surface selective dissolution
410	18	12	All-cellulose nanocomposites by surface selective dissolution of bacterial cellulose
89	17	13	Novel all-cellulose ecocomposites prepared in ionic liquids
74	0.75	14	Preparation of all-cellulose composite by selective dissolving of cellulose surface in PEG/NaOH aqueous solution
76	13	15	A non-solvent approach for high-stiffness all-cellulose biocomposites based on pure wood cellulose
128.5	4.8	16	Discrimination of matrix–fibre interactions in all-cellulose nanocomposites
65	1.5	17	Self-reinforced cellulose nanocomposites
52	3.7	18	Green composite films composed of nanocrystalline cellulose and a cellulose matrix regenerated from functionalized ionic liquid solution
188	17.5	19	Direct Fabrication of all-Cellulose Nanocomposite from Cellulose Microfibers Using Ionic Liquid-Based Nanowelding
140	5.6	20	Novel all-cellulose composite displaying aligned cellulose nanofibers reinforced with cellulose nanocrystals
46	0.86	21	All-cellulose composite laminates
70.16	2.45	22	All-cellulose composite laminates
78	7.2	23	All-cellulose composites prepared from flax and lyocell fibres compared to epoxy-matrix composites
175.6	12.5	24	Influence of Magnetic Field Alignment of Cellulose Whiskers on the Mechanics of All-Cellulose Nanocomposites
34	4.6	25	All-cellulose composites prepared from flax and lyocell fibres compared to epoxy-matrix composites
170	13.6	26	Orientation and deformation of wet-stretched
165.4	11.8	27	Deformation micromechanics of all-cellulose nanocomposites: comparing matrix and reinforcing components
137.1	9.8	28	Deformation micromechanics of all-cellulose nanocomposites: comparing matrix and reinforcing components

20	0.05	29	All-cellulose and all-wood composites by partial dissolution of cotton fabric and wood in ionic liquid
12	0.14	30	Properties of All-Cellulose Composite Films from Coconut Shell Powder and Microcrystalline Cellulose
135	8.1	31	All-Cellulose Nanocomposites Reinforced with in Situ Retained Cellulose Nanocrystals during Selective Dissolution of Cellulose in an Ionic Liquid
650	11	32	All-cellulose composites with ultra-high mechanical properties prepared through using straw cellulose fiber
126.2	5.6	33	Production of regenerated nanocomposite fibers based on cellulose and their use in all-cellulose composites
109	3.56	34	All-cellulose composite isolated from oil palm empty fruit bunch
140	12.8	35	All-cellulose nanocomposite film made from bagasse cellulose nanofibers for food packaging application
16	3.8	36	All-cellulose composites from alfa and wood fibers
13.9	4.2	37	All-cellulose composites from alfa and wood fibers
144	5.5	38	All-Cellulose Composites by Partial Dissolution of Cotton Fibres
77.7	7.3	39	Parametric optimization of the processing of allcellulose composite laminae
92	4	40	Solvent infusion processing of all-cellulose composite material
64.9	5.5	41	Potential of all-cellulose composites in corrugated board applications: Comparison of chemical pulp raw materials
45	1.7	42	All-cellulose composite laminates with low moisture and water sensitivity
167	6.2	43	Cellulose nanofibrils improve the properties of all-cellulose composites by the nano-reinforcement mechanism and nanofibril-induced
44.2	1.8	44	Effect of pressure on structure and properties of lyocell fabric-based all-cellulose composite laminates
4.2	102.6	45	A comparative study on lyocell-fabric based all-cellulose composite laminates produced by different processes
